# Cationic lipid-assisted nanoparticles for simultaneous delivery of CD47 siRNA and R848 to promote antitumor immune responses

**DOI:** 10.3389/fphar.2023.1142374

**Published:** 2023-03-31

**Authors:** Simin Li, Yichang Chen, Ruolin Ma, Ye Du, Bing Han

**Affiliations:** Breast Surgery Department, General Surgery Center, First Hospital of Jilin University, Changchun, China

**Keywords:** TNBC, siCD47, R848, PEG-PLGA nanoparticles, tumor immunotherapy

## Abstract

**Introduction:** Triple-negative breast cancer (TNBC) usually has a poor prognosis. Although the immunotherapy of TNBC has achieved great progress, only a few patients can benefit from the current treatment. CD47 is widely expressed on the surface of TNBC cells and may become an immune checkpoint for TNBC treatment. Nevertheless, increasingly more attention has been paid to systemic side effects since the ubiquitous expression of CD47 on normal cells. The toll-like receptor (TLR) agonist resiquimod (R848) can activate dendritic cells (DCs) and promote the maturation of immune cells in the tumor microenvironment, which further enhances the tumor inhibition ability of the immune system and synergizes with CD47 small interfering RNA (siRNA) for TNBC therapy. However, ideal delivery platforms such as nanocarriers are still needed because its weakness of hydrophobicity.

**Methods:** In order to improve efficacy and reduce toxicity, R848 and siCD47 were entrapped in amphiphilic PEG-PLGA nanoparticles by double emulsification and stable nanoparticles NP/R848/siCD47 were generated to investigate their anti-tumor effects in a TNBC tumor-bearing mouse model.

**Results:** Here, we show that PEG-PLGA nanoparticles are effective nanocarriers that can safely and effectively deliver siCD47 and R848 to tumor tissue, as demonstrated by retarded tumor growth. Mechanistically, downregulation of CD47 expression and activation of DCs took part in promoting the immune response of cytotoxic T cells (CTLs). Meanwhile, a decrease of myeloid-derived suppressor cells (MDSCs) and tumor-associated macrophages (TAMs) indicated the modulating of the tumor immune microenvironment.

**Discussion:** To our best knowledge, our study pioneered co-delivery system for hydrophilic siCD47 and hydrophobic R848. It can maximize break tumor immune escape caused by CD47 and simultaneously enhance antigen presentation by activating DCs for effector T cell killing while regulating the tumor microenvironment as expected. Not only does it conform to the reports of previous basic research, but also it can break the bottleneck of their clinical application hopefully. Collectively, our findings could lay the foundation for future therapeutic strategies of TNBC.

## Graphical Abstract

**Figure:**
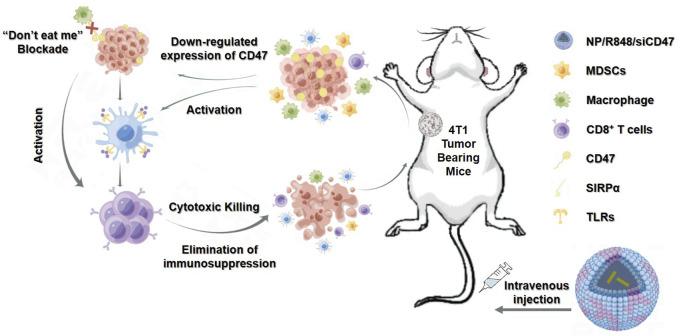
The PEG-PLGA nanoparticles effectively delivered R848 and CD47 siRNA into tumor cells, resulting in simultaneous activation of DCs and downregulation of CD47 expression on tumor cells, thereby enhancing antitumor immune responses by T cells.

## Introduction

Triple-negative breast cancer (TNBC) is a molecular subtype which accounts for approximately 15% of all invasive breast cancers with negative expression of the estrogen receptor (ER), progesterone receptor (PR), and human epithelial growth factor receptor-2 (HER-2) (Bianchini et al., 2016). TNBC is characterized by poor prognosis, high invasion, high metastatic potential, and high mortality (Foulkes et al., 2010). Endocrine therapy and molecular targeted therapy are ineffective for TNBC due to the lack of ER, PR, and HER-2 expression (Gluz et al., 2009; Sinha et al., 2021).

Immune surveillance can usually identify and eliminate mutated cells, thus preventing the occurrence and development of tumors. Tumor is driven by immune escape. Tumor immunotherapy can be broadly divided into tumor antigen-specific and non-specific categories. Non-specific immunotherapy includes non-specific immune stimulation and immune checkpoint blocking, while the specific category refers to multiple tumor vaccines and adoptive cell transfer therapy (Zhang and Zhang, 2020). Tumor immunotherapy shows unique advantages (Darvin et al., 2018). It can inhibit tumor growth and break immune tolerance by activating the immune cells and enhancing the anti-tumor immune response. The emergence of immune checkpoint inhibitors (ICIs) is a revolutionary milestone in the field of tumor immunology, which shows a significant therapeutic effect in many solid tumors, such as lung cancer (Jia et al., 2020), pancreatic cancer (Bear et al., 2020), and melanoma (Sockolosky et al., 2016), and may be expanded for the potential treatment of TNBC. CD47 is a “self-recognition molecule” on the cell surface that is widely expressed in human cells which can bind to a signal regulatory protein on phagocytes α (SIRP α), followed by activating protein tyrosine phosphatase (PTP) sending out “self”-inhibition signals to escape the phagocytosis of macrophages and DCs, avoiding the cytotoxicity of T cells under innate immune surveillance, and finally causing immune evasion (Chao et al., 2010). Small interfering RNA targeting *CD47* gene (CD47 siRNA, siCD47) silences CD47 expression, promotes the recognition and clearance of tumor cells by the immune system, and enhances the anti-tumor immune response dependent on T cells. However, siRNA is easy to be cleared by RNase enzyme. Its physiological characteristics limit its use *in vivo* (Elbashir et al., 2001; Gallas et al., 2013). At the same time, the TLR agonist resiquimod (R848) activates DC in the tumor microenvironment, promotes the maturation of tumor microenvironment immune cells, and further enhances the anti-tumor effect of the immune system. R848 can synergistically improve the therapeutic effect of siCD47 on TNBC. However, its hydrophobicity also affects utilization *in vivo* (Cherfils-Vicini et al., 2010; van Haren et al., 2016; Beesu et al., 2017). With the development of nanotechnology, the nanomedicine delivery system enables drugs to enrich the tumor tissue and improves the drug uptake rate of target cells. Nanocarriers could protect the drug activity, reducing the non-specific reaction and “off-target” effects of gene drugs *in vivo* and, meanwhile, avoiding the toxic side effects of hydrophobic drugs (Raposo et al., 2020). Compared to the delivery of different agents separately, co-delivery has the advantages of ensuring the efficient enrichment of two drugs in the tissue simultaneously and guaranteeing the two drugs being delivered to the cells, according to the preset proportion. Additionally, the co-delivery system permits the access of context-specific multiple targets and releases “on demand” at the tumor site (Zhang et al., 2015; Pan et al., 2020). Moreover, this amphiphilic copolymer also suits the co-delivery of other hydrophilic and hydrophobic drugs for overcoming single-drug resistance, reduces dosage and administration frequency of each agent, and avoids systemic toxicity (Kaur et al., 2016). Application of nanoparticles enhances the anti-tumor effect and greatly increases drug safety (Zhao et al., 2011; Willingham et al., 2012). Our study aimed at inhibiting the expression of *CD47* gene in the tumor cells to reduce immune escape, activating antigen-presenting cells (APCs), and eliminating the tumor immunosuppressive microenvironment through the construction of siCD47 and R848-loaded nanoparticles. The anticipated anti-tumor effect may provide new insights into TNBC immunotherapy.

## Materials and methods

### Reagents

Dulbecco’s modified Eagle’s medium (DMEM), PBS, penicillin/streptomycin, l-glutamine, fetal bovine serum (FBS), Aqua Dead Cell Stain Kit, Lipofectamine 2000, TRIzol, and collagenase type IV were purchased from Thermo Fisher Scientific (Waltham, MA, United States). The RBC lysis buffer was purchased from Solarbio (Beijing, China). siRNA-targeting mouse CD47 mRNA (antisense strand, 5’-UGGUGAAAGAGGU-CAUUCCdTdT-3’) and negative control siRNA with a scrambled sequence (antisense strand, 5’-ACGUGACACGUUCGGAGAAdTdT-3’) were synthesized by Suzhou Biosyntech Co. Ltd. (Suzhou, China). Anti-CD47 antibody was purchased from Santa Cruz Biotechnology (Texas, United States). The Click-iT Plus TUNEL Assay was performed for *in situ* apoptosis detection; the Alexa Fluor 647 dye was purchased from Thermo Fisher Scientific (MA, United States).

### Cell culture

The murine breast cancer 4T1 cells were obtained from the American Type Culture Collection and were maintained in DMEM (Carlsbad, CA, United States) supplemented with 10% FBS (Waltham, MA, United States) and 1% penicillin with streptomycin (complete DMEM) at 37°C in a 5% CO_2_ humidified atmosphere. Bone marrow-derived dendritic cells (BMDCs) were generated by flushing bone marrow (BM) cells in the femur and tibia of BALB/c mice, followed by culturing for 7 days in RPMI 1640 medium supplemented with 10% FBS, GM-CSF (10 ng/mL), and mouse IL-4 (10 ng/mL).

### Animals and the tumor model

Female BALB/c mice and BALB/c nude mice (6–7 weeks old) were purchased from Charles River Laboratories (Beijing, China) and raised in a specific pathogen-free environment with free access to food and water. All animals received care in compliance with the guidelines outlined in the Guide for the Care and Use of Laboratory Animals. All procedures were approved by the Jilin University Animal Care and Use Committee. To establish the tumor-bearing mouse model, 4T1 cells (5 × 10^5^) were suspended in 100 μL of PBS and were administered by subcutaneous injection into the armpit of the mice. Tumor volume (cubic millimeter) was determined by measuring the length (L) and width (w) and calculated as V = lw^2^/2.

### Preparation and characterization of CD47 siRNA- and R848-loaded nanoparticles

An amount of 2.5 mg of the polylactic acid/glycolic acid copolymer (PEG-PLGA) was dissolved in 0.4 mL trichloromethane (CHCL_3_, 10% wt/vol), and 0.3 mg DOTAP was dissolved in 0.1 mL CHCL_3_. Then, 5 g of resiquimod (R848) was dissolved in 5 L dichloromethane regarded as the oil phase, and 25 uL of the siCD47 solution (plasmid concentration, 8 ng/μL) was added to the organic phase regarded as the water phase, that is, the volume ratio of PEG-PLGA and siCD47 solution was 20:1, and the mixture was fully mixed. The liquid colostrum (W/O) was prepared by sonication (100 W, 60 s) on ice using a Vibra-Cell VCX130 (Sonic & Materials Inc., Newtown, United States). A measure of 5 mL of ddH_2_O added to the colostrum, and the ice bath was ultrasonicated with the same power for 1 min to form W/O/W complex emulsion solution. The compound milk was poured into a circular narrow mouth flask and then slowly rotated to remove dichloromethane using a water bath at 37°C. The supernatant was centrifuged at 3000 r/s for 90 min, and then, the supernatant was discarded. After resuspension with an appropriate amount of ddH_2_O, the supernatant was centrifuged once again. Then, it was resuspended with 1 mL ddH_2_O, and the bacteria were filtered with a 450-nm filter. The isosmotic state should be adjusted for *in vivo* and *in vitro* experiments. The particle size and zeta potential of nanospheres were measured by using a Malvern ZS90 particle size analyzer. The morphologies of these nanoparticles were observed using a scanning electron microscope operated at 3 kV (SU8000; Hitachi, Tokyo, Japan).

### Loading efficiency of siCD47

NP samples after rotary evaporation were collected for detecting the content of free non-entrapped siRNA. NP/R848/siCD47 and 10 μL free siCD47 with the same concentration of siCD47 were mixed with a 1 μL loading buffer and added to different pores of GelRed dried 2% agarose gel for gel electrophoresis (80 mV, 10 min). The encapsulation efficiency of siCD47 in NP/R848/siCD47 was calculated by (total siRNA added—free non-entrapped siRNA) divided by the total siRNA added.

### Stability of NP/R848/siCD47 in the serum

The susceptibility to serum degradation of free or complex siRNA within NP/R848/siCD47 was evaluated with the agarose gel electrophoresis assay and particle size. NP/R848/siCD47 and equivalent amount of free siRNA were incubated in PBS, containing 25% serum at 37°C for 0, 2, 6, or 24 h. Then, the size of nanoparticles was monitored using a dynamic light scattering (DLS) technique. Heparin was added to these samples at a heparin/siRNA (w/w) ratio of 5:1 to release the complex siRNA cargo. After 30 min of incubation, each sample was loaded on the 1% agarose gel and allowed to run at 100 V for 30 min for further detection.

### Cellular uptake study

The lipophilic dye 1,1’-dioctadecyl-3,3,3’,3’-tetramethylindodicarbocyanine, 4-chlorobenzenesulfonate salt (DiD) was used for detecting the tracing of nanoparticles. 4T1 cells and BMDC were seeded in 24-well plates at a density of 1 × 10^5^ cells per well with 500 μL of complete DMEM and incubated overnight at 37°C in an incubator with 5% CO_2_. Nanoparticles loaded with PBS or 20 μL NP/DiD in 500 μL of DMEM were added to each well. After incubation at 37°C for 2 h, the cells were analyzed by flow cytometry.

### *In vitro* gene silencing with NP/siCD47

4T1 cells were seeded into 24-well and 6-well plates and cultured overnight at 37°C. The cells were treated with PBS or NP at a siRNA dose of 50 nM. Lipofectamine 2000-transfected siCD47 at a siRNA dose of 100 nM was used as positive control. After 24 and 48 h of incubation, the cells were harvested and the expression of CD47 was evaluated by qPCR, flow cytometry, and Western blotting.

### Quantitative real-time PCR analysis

Total RNA from NP/R848/siCD47 and other control-treated 4T1 cells were extracted using TRIzol. cDNA was synthesized using the HiScript II Q RT SuperMix for qPCR (+gDNA wiper) (Vazyme, Nanjing, China). For the purpose of determining the expression level of CD47 mRNA, cDNA was subjected to qPCR analysis targeting CD47 and β-actin using the ChamQ Universal SYBR qPCR Master Mix (Vazyme, Nanjing, China). The quantitative real-time PCR was performed using the StepOnePlus Real-Time PCR system (Applied Biosystems, Foster City, CA, United States), and cycle threshold (Ct) values were calculated with StepOne software (Applied Biosystems, Foster City, CA, United States). The primers used for mouse CD47 and β-actin models were CD47-forward: 5′-CAA​AAC​TAC​CAC​ATT​CCC​TAC​CC -3′; CD47-reverse: 5′-ACA​TAC​AAA​CAC​GCC​CAC​AAA​C-3′; β-actin-forward: 5′- TTCAACACCCCAGCCATG -3′; β-actin-reverse: 5′- CCT​CGT​AGA​TGG​GCA​CAG​T -3′.

### Gel electrophoresis and Western blotting

Samples were collected and centrifuged at 1,000 × g for 10 min at 4°C to obtain the supernatant, and the protein content of the supernatant was determined using the BCA protein assay kit (Beyotime Institute of Biotechnology). Equal quantities of protein were electrophoresed on 8%–12% sodium dodecyl sulfate–polyacrylamide gels based on the molecular weight of the target protein and transferred to PVDF membranes (EMD Millipore). The membranes were then blocked with 5% skimmed milk in PBS for 2 h at room temperature and incubated overnight at 4°C with primary antibodies. The membranes were washed with the PBS 0.1% Tween 20 (PBS-T) buffer for 30 min at 25°C prior to incubation with horseradish peroxidase-conjugated goat anti-rabbit IgG (1:1,000) at 25°C for 2 h. Membranes were subsequently washed with the PBS-T buffer, and immunoreactive proteins were visualized on a chemiluminescence developer (ChemiScope 5300; Clinx Science Instruments, Co., Ltd.) with an enhanced chemiluminescence reagent (P10300; NCM Biotech).

### Tumor accumulation and tissue distribution of NP/R848/siCD47

After 12 days with respect to tumor cell inoculation, the tumor-bearing mice were administered with NP/R848/siCD47, NP/DiD, or PBS by intravenous injection. The doses of siRNA were 5 mg/kg each. Fluorescent image acquisition was performed at 1, 2, and 24 h after injection using the Xenogen IVIS Lumina system (Caliper Life Sciences, United States). The tissues containing tumor, lymph node, and other organs of these mice were harvested and imaged 24 h after injection for observing the distribution of NP/R848/siCD47. The results were analyzed using Living Image^®^ 3.1 software (Caliper Life Sciences). The tumors and spleens were prepared as single cells, and flow cytometry was used to quantitatively analyze the percentage of DiD-positive cells in tumor and DCs.

### Treatment of tumor-bearing mice with NPs

Tumor-bearing BALB/c mice were prepared by injection of 3 × 10^5^ 4T1 cells into the mammary fat pad and were given NP/R848/siCD47, NP/R848/siNC, NP/siCD47, NP/siNC, or PBS by intravenous injection 10 times. The doses of siRNA for each injection were 1 × 10^5^ nmol/kg, respectively. Tumor volumes and the mouse weights were measured every day. The day after the last treatment, tissues containing tumor, lymph node, and other organs of these mice were excised for preparation of single-cell suspension for flow cytometry assay or fixed with 4% paraformaldehyde and embedded in paraffin for HE staining and TUNEL assay. Tissue sections were visualized under a laser scanning confocal microscope (Olympus FV1000, Tokyo, Japan).

### Statistical analysis

Statistics were performed on GraphPad Prism 8, and unpaired Student’s t tests or one-way analysis of variance (ANOVA) were used to compare the paired and unpaired analyses. The statistical evaluation of mouse survival was performed using the Kaplan–Meier method and the log-rank test. *p* values <0.05 were considered statistically significant.

## Results

### Characterization of nanoparticles

**FIGURE 1:**
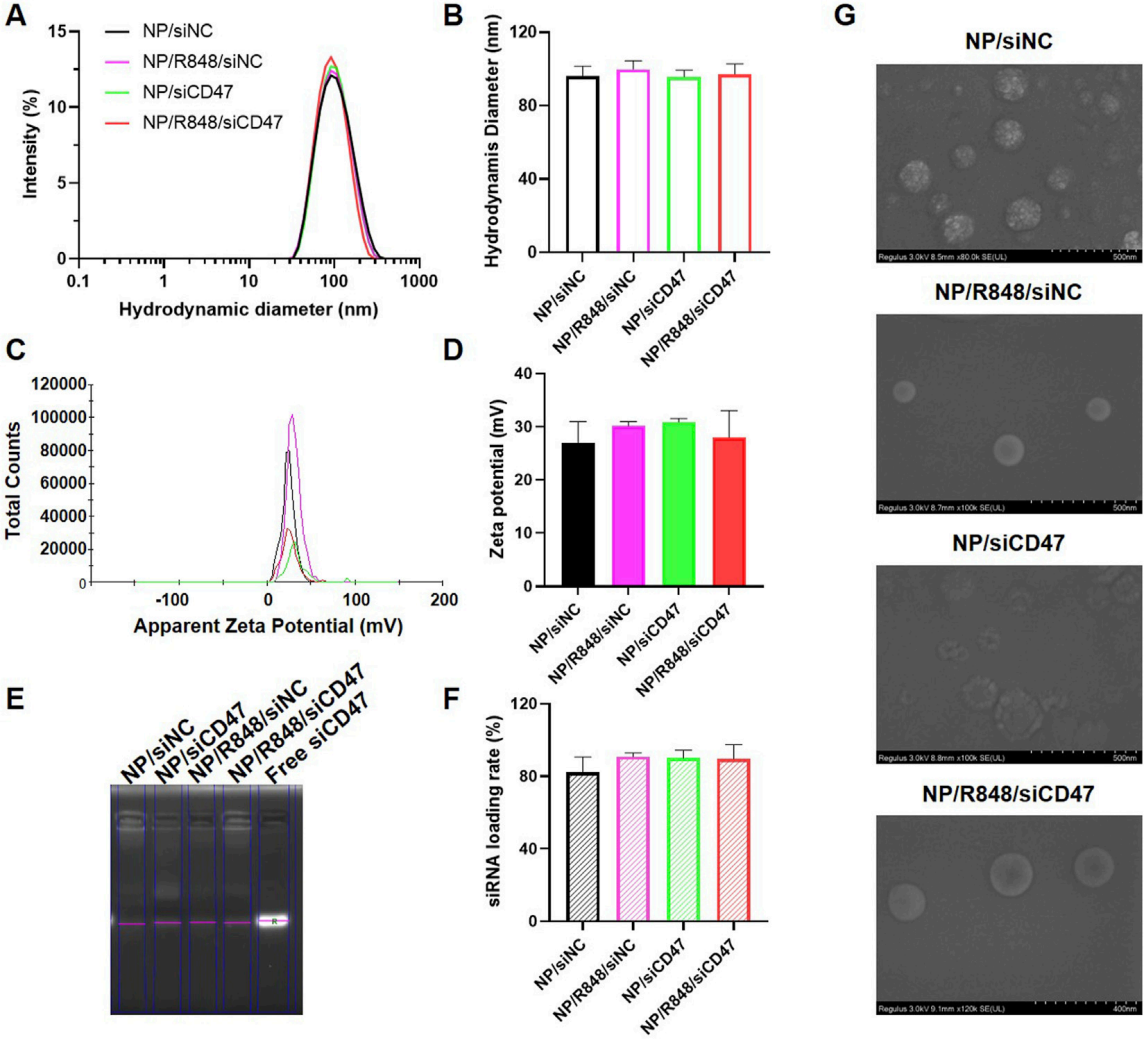
Characterization of four nanomedicines. **(A,B)** Hydrodynamic diameters of NP/siNC, NP/siCD47, NP/R848/siNC, and NP/R848/siCD47. **(C,D)** Zeta potential of NP/siNC, NP/siCD47, NP/R848/siNC, and NP/R848/siCD47. **(E)** RNA agarose gel electrophoresis. The un-encapsulated siRNA during the preparation of the NP/siNC, NP/siCD47, NP/R848/siNC, and NP/R848/siCD47 was measured by RNA agarose gel electrophoresis. **(F)** siRNA-loading efficiencies of NP/siNC, NP/siCD47, NP/R848/siNC, and NP/R848/siCD4. **(G)** Scanning electron microscopic (SEM) image of NP/siNC, NP/siCD47, NP/R848/siNC, and NP/R848/siCD47. The scale bar is shown in the figure. Data were presented as mean ± SEM (*n* = 5).

The hydrodynamic diameters and surface zeta potential of four nanodrugs were detected by dynamic light scattering (DLS). As shown in Figures 1A–D, the hydrodynamic diameters were 96.0 ± 12.5 nm for NP/siNC, 99.8 ± 10.2 nm for NP/R848/siNC, 95.8 ± 8.1 nm for NP/siCD47, and 97.3 ± 12.2 nm for NP/R848/siCD47. The zeta potentials were 27.0 ± 7.8 mV for NP/siNC, 30.2 ± 1.6 mV for NP/R848/siNC, 30.9 ± 1.5 mV for Np/siCD47, and 28.0 ± 8.8 mV for NP/R848/siCD47. The siRNA-loading efficiency was calculated by RNA agarose gel electrophoresis (Figures 1E, F). The siCD47 encapsulation efficiencies of NP/siCD47 and NP/R848/siCD47 were approximately 90.1% and 89.7%, respectively. These findings indicated the success of loading. Scanning electron microscopy revealed that all these nanoparticles displayed a uniform spherical shape (Figure 1G). We also investigated the stability of NP/R848/siCD47 under physiological conditions. Obvious degradation of siRNA was noticed in free siRNA after 6 h of incubation in 25% FBS, while no significant change was observed in siRNA encapsulated by NP/R848/siCD47 within 24 h. It implied that our nanoparticles could protect the siRNA from enzymatic degradation (Supplementary Figure S1A). Meanwhile, as shown in Supplementary Figure S1B, the particle size of NP/R848/siCD47 showed statistically no difference up to 24 h after incubation in FBS *in vitro*, reflecting that NP/R848/siCD47 has good stability in the serum. Here, we explain the preparation process of nanoparticles in Supplementary Figure S1C.

### NP/R848/siCD47 downregulates CD47 expression and promotes maturation of BMDCs *in vitro*

**FIGURE 2:**
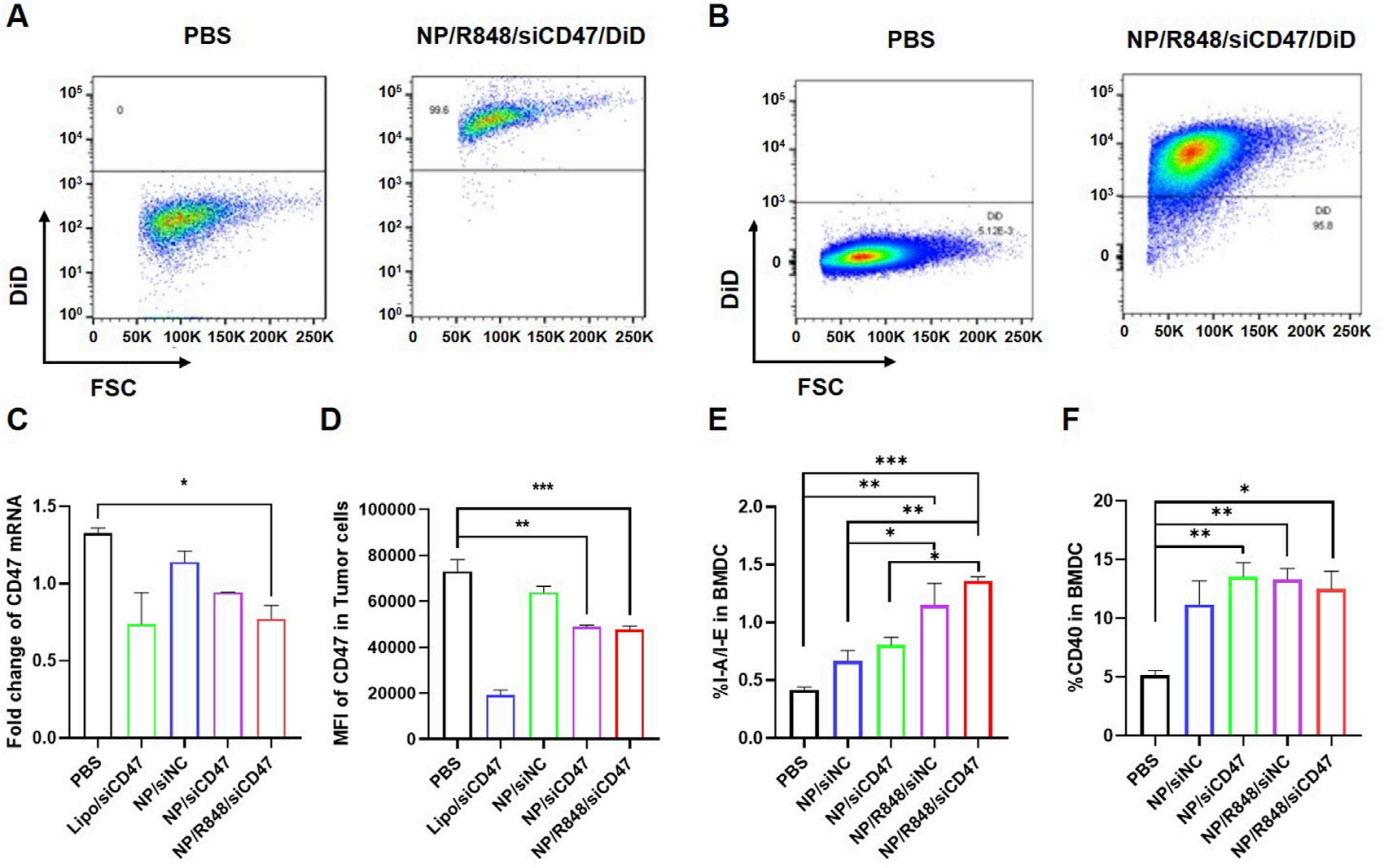
Phagocytosis of NP/R848/siCD47/DiD by 4T1 cells and BMDCs induced *in vitro*. **(A,B)** Representative flow cytometry demonstrating the DiD fluorescence in 4T1 cells and BMDCs induced *in vitro* after being cultured with PBS and NP/R848/siCD47/DiD for 2 h. **(C)** mRNA levels of CD47 in 4T1 cells after incubation with PBS, NP/siNC, NP/siCD47, and NP/R848/siCD47 for 24 h were assayed by qPCR. **(D)** Flow cytometry analysis relative to MFI of the surface expression of CD47 on 4T1 cells upon treatment with PBS, NP/siNC, NP/siCD47, and NP/R848/siCD47 for 48 h. **(E,F)** Surface expression of CD40 and I-A/I-E in BMDCs measured by flow cytometry. Data were presented as mean ± SEM (*n* = 3). (*, *p* < 0.05; **, *p <* 0.01; ***, *p* < 0.001).

A nanoparticle containing a hydrophobic fluorescent dye (DiD) was prepared by double emulsification to detect whether it could be uptaken by 4T1 cells and BMDCs *in vitro*. 4T1 cells and BMDCs induced on the seventh day were incubated with PBS or DiD nanoparticles for 2 h, respectively, and then, the fluorescence intensity for DiD was measured by flow cytometry. As revealed in Figure 2, almost all 4T1 cells (Figure 2A) and BMDCs (Figure 2B) displayed DiD-positive fluorescence signals compared to those of the PBS group.

Subsequently, we assessed the ability of NP/R848/siCD47 to downregulate CD47 expression in mouse breast cancer cells. 4T1 tumor cells were treated with PBS, NP/siNC, NP/siCD47, NP/R848/siNC ,and NP/R848/siCD47 for 24 h (50 nM for siRNA). The mRNA expression of CD47 was assessed by qPCR. Compared with other control nanoparticles, CD47 expression was downregulated by NP/siCD47 and NP/R848/siCD47 (Figure 2C). This result is consistent with the observed trend obtained by flow cytometry and Western blotting (Supplementary Figure S2). The expression of surface CD47 in 4T1 cells was reduced after incubation with NP/siCD47 and NP/R848/siCD47 for 48 h (50 nM for siRNA) (Figure 2D).

In addition, compared to PBS, NP/R848/siNC and NP/R848/siCD47 treatments significantly enhanced the activation and maturation of BMDCs (Figures 2E, F). These data suggest that NP/R848/siCD47 may hold great potential in tumor immunotherapy by inhibiting the expression of *CD47* gene in tumor cells so as to reduce tumor immune evasion while activating APCs and contributing to tumor suppression *in vivo*.

### Nanoparticles accumulating in the tumor tissue *in vivo*

**FIGURE 3:**
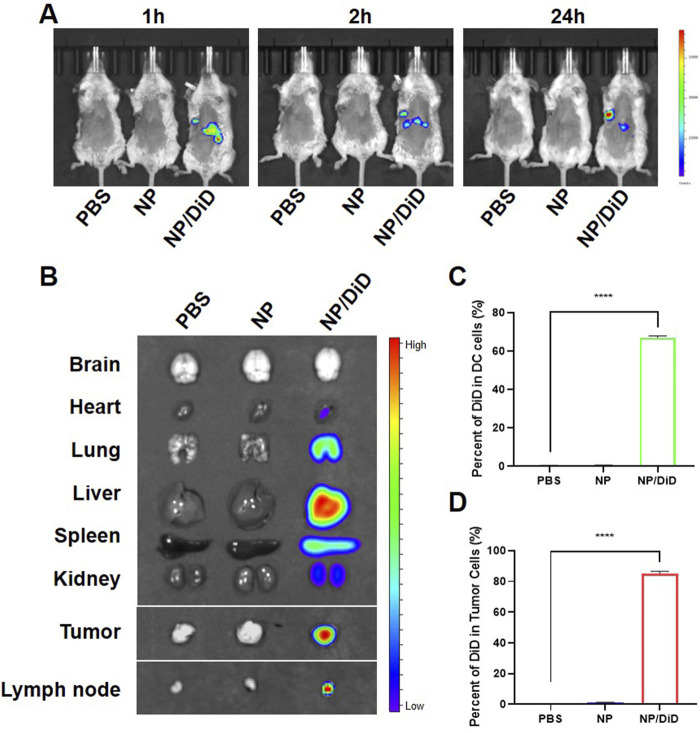
Biodistribution of drug-loaded nanoparticles in tumor-bearing mice. **(A)** *In vivo* fluorescent imaging of tumor-bearing mice after intravenous injection of NP/R848/siCD47/DiD at different time points. **(B)** *Ex vivo* fluorescent imaging of the tumor and other organs. **(C,D)** Percentage of DiD^+^ cells in DCs and CD45^−^ tumor cells isolated from mice injected with PBS, NP/R848/siCD47, or NP/DiD. Data were presented as mean ± SEM (*n* = 3). ****, *p* < 0.0001 by multiple one-way ANOVA.

In order to determine whether NP/R848/siCD47 nm drugs can perform tumor-targeted delivery, we injected PBS, NP/R848/siCD47, and NP/DiD into tumor-bearing mice *via* tail vein, respectively. We found that NP/DiD was accumulated in a time-dependent manner. As illustrated in Figure 3A, the fluorescent signal for DiD was found in the tumor region as early as 1 h after intravenous (i.v.) injection with NP/DiD and was also stably detectable at 24 h. Significant accumulation of NP/DiD was also found in the tumor, heart, lung, liver, spleen, kidney, and draining lymph node (Figure 3B). Flow cytometric analysis illustrated that approximately 60% of CD11b^+^CD11c^+^ DCs and 80% of CD45^−^ tumor cells were DiD^+^ 24 h after i.v. injection with NP/DiD (Figures 3C, D). All the aforementioned results showed a high efficacy of this PEG-PLGA-based delivery system for the simultaneous delivery of hydrophobic and hydrophilic drugs into tumor cells and immunocytes *in vivo*.

### NP/R848/siCD47 inhibits tumor growth safely and effectively *in vivo*

**FIGURE 4:**
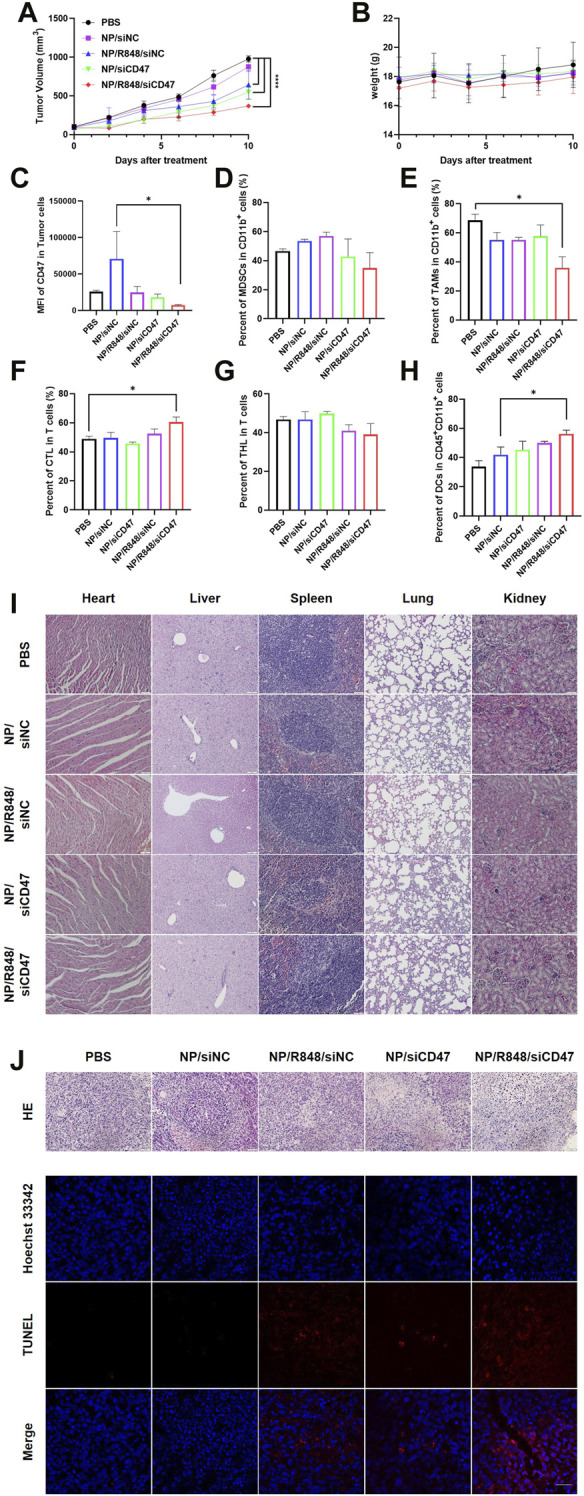
NP/R848/siCD47 significantly inhibited tumor growth and enhanced anti-tumor immune responses *in vivo*. **(A)** Tumor growth curves of different groups of mice after various treatments **(B)** Body weights of 4T1 tumor-bearing mice in the tumor inhibition experiment of different groups of mice after various treatments. **(C)** Flow cytometry analysis of CD47 expression in tumor tissues treated with PBS, NP/siNC, NP/siCD47, NP/R848/siNC, and NP/R848/siCD47 at the end of the tumor-inhibition experiment. **(D)** Percentage of myeloid-derived suppressor cells (MDSCs) (CD11b^+^Gr-1^+^). **(E)** Percentage of tumor-associated macrophages (TAMs) (CD11b^+^F4/80^+^). **(F,G)** Percentage of cytotoxic T cells (CD3^+^CD8^+^) and helper T cells (CD3^+^CD4^+^). **(H)** Percentage of DCs (CD11b^+^CD11c^+^) in CD45^+^CD11b^+^ myeloid immune cells. **(I)** Microscopic image of HE staining of the main organ paraffin section. The scale bar is shown in the figure. **(J)** Microscopic image of HE staining of the tumor tissue paraffin section. The scale bar is shown in the figure. Fluorescence diagram of the TUNEL assay in tumor tissues treated as described in **(C)**. The scale bar is 20 μm. Data were presented as mean ± SEM (*n* = 3). (*, *p* < 0.05; **, *p* < 0.01; ***, *p* < 0.001; ****, *p* < 0.0001).

In order to investigate the tumor therapeutic effects of siCD47 and R848 nanoparticles, a 4T1 tumor-bearing mouse model was randomly divided into five groups, namely, NP/siNC, NP/siCD47, NP/R848/siNC, NP/R848/siCD47, and PBS. Each nanoparticle was injected (i.v.) daily for 11 days at concentrations of 2.5 nmol/20 g for siCD47, respectively. Except the NP/siNC group, other treatment groups had obvious tumor inhibition effects compared with the PBS group. Of note, NP/R848/siCD47 exhibited the greatest anti-cancer effects compared with other groups (Figure 4A). The result of microstructure examination indicated that the tumor growth rate was negatively correlated with cell necrosis and interstitial fibrosis. Meanwhile, tumor cell apoptosis in the treatment group also increased through confocal microscopy of the terminal deoxynucleotidyl transferase dUTP nick end labeling (TUNEL) staining section (Figure 4J). The mice in all groups survived throughout the experimental period with no body weight loss or other signs indicative of toxicity (Figure 4B). It was also found by observing HE staining of the paraffin section under the microscope that there was no obvious injury in the main organs of mice after the treatment (Figure 4I), which further confirmed the safety of nanoparticles. Flow cytometry analysis of the tumor tissue revealed that the treatment with NP/siCD47 and NP/R848/siCD47 weakened the expression of CD47 in line with the *in vitro* experiment (Figure 4C). This evidence suggested that the antitumor effect was closely associated with downregulation of CD47 expression in tumor cells and could be further promoted, following immune response. We next analyzed the tumor-infiltrating immune cell composition, with a focus on myeloid-derived suppressor cells (MDSCs), tumor-associated macrophages (TAMs), and T cells. In groups that contain siCD47, we observed that MDSCs had a decreased tendency than those injected within the control groups, although without statistical significance (Figure 4D). Meanwhile, the proportion of TAMs decreased significantly in the NP/R848/siCD47 groups, as expected (Figure 4E), suggesting that the inhibitory medullary microenvironment was gradually broken down. Furthermore, the ratio of cytotoxic T cells in CD11b^−^CD3^+^ T cells was higher in mice treated with NP/R848/siCD47 than those in the PBS group (Figures 4F, G). Remarkably, the percentage of DCs in CD45^+^CD11b^+^ cells significantly increased in the NP/R848/siCD47-treated groups (Figure 4H). These results confirmed that the efficacy of tumor resistance correlated with a significant increase in CD8^+^ T cells and APCs, which promotes immune recognition and surveillance.

## Discussion

TNBC is a highly invasive molecular subtype of breast cancer, which is usually associated with poor prognosis. Treatment of TNBC is limited due to its resistance to chemotherapy. In recent years, the application of immune checkpoint inhibitors (ICB) has brought new hope for the treatment of TNBC. However, TNBC is traditionally immunologically “cold” with weak immunogenicity and poor DC-stimulating activity; thus, tumor cells often escape from immune surveillance and cause ICB resistance.

CD47 is a transmembrane protein widely expressed but often overexpressed in various tumor types including breast cancer. Tumor cell-associated CD47 has been shown to promote proliferation, metastatic potential, and drug resistance, and regain tumor-initiating ability and EMT. It has been reported that HIF-1 directly activated transcription of the *CD47* gene in hypoxic breast cancer cells (Soto-Pantoja et al., 2014). In translational studies, analysis of datasets derived from breast cancer patients also revealed that high CD47 mRNA expression levels are correlated with mortality and poor prognosis of patients. The TNBC (MDA-MB-231) treatment with anti-CD47 mAb showed decreased proliferation and inhibited expression of the stem cell transcription factor (Van et al., 2006). One essential and well-studied function of CD47 related to tumor development is preventing phagocytosis *via* ligating with signal regulatory protein-alpha (SIRPα) on the surrounding phagocytes and evading destruction by using the innate and adaptive immune system (Grimbert et al., 2006). Interference with CD47–SIRPα interactions by antagonistic antibodies or CD47 knockdown obviously enhances the *in vitro* killing of trastuzumab-treated Her-2^+^ breast cancer cells by phagocytes. Correspondingly, the response to trastuzumab therapy in breast cancer patients appears related to CD47 expression of the cancer cell (Avice et al., 2000). These results led to the development of several other SIRPa–CD47-blocking agents and obtained efficacy *in vitro* and in preclinical studies against different human tumors. Some early-phase clinical trials targeting the CD47/SIRPα axis are now being tested. In addition, T-cell-mediated adaptive immune response acts crucially in anti-CD47 blockade-triggered tumor control (Liu et al., 2015). T-cell activation is decreased in response to reduced tumor cell ingestion by antigen-presenting cells (APCs) (Bian et al., 2016). Furthermore, activation of CD47 on naive T cells promotes the formation of Tregs (Heil and Vega-Munoz, 2019) and inhibits formation of T helper 1 effector cells (Miller et al., 1999). Intriguingly, it was previously found that dendritic cells—not macrophages—appeared to play a more valuable role for CTL cross-priming and anti-tumor therapy (Vacchelli et al., 2013). These lines of evidence confirm our research results. Accumulating evidence suggests that ICB of CD47 is a promising novel way to cure cancer. However, the possibility of attack against healthy cells is more worthy to be concerned. Some patients develop pronounced side effects including serious autoimmunity. Patients specifically under infection or chronic inflammatory conditions may become severely anemic upon CD47 blockade (Lu et al., 2019a). Given the ubiquitous expression of CD47 on normal cells, tumor-specific delivery of CD47 blockade would generate better anti-tumor effects with fewer side effects than systemic administration. We use the nanodrug delivery system to deliver CD47 siRNA to tumor tissues and tumor cells, which can effectively improve the targeted regulation of CD47 molecules in tumor cells.

The tumor invasion microenvironment plays a key role in the occurrence, development, and metastasis of the tumor. Although DC cells can be activated by blocking the function of the CD47 molecule, the degree of activation is still limited. Using immunomodulators to enhance the activation of DC cells while silencing the expression of the CD47 molecule in tumor cells can further improve the anti-tumor immune response. TLRs are membranous or endosomal pattern recognition receptors (PRRs), which is important in innate immunity (Lu et al., 2019b). One of the TLR family members, TLR7/8, has become a promising target since reacting to single-stranded RNA rich in uridine and guanosine. The TLR7 agonist imiquimod has been approved by the FDA for topical treatment of actinic keratosis and genital warts as early as in 1997 ((Rao et al., 2019)). R848, as a derivative of imiquimod, can APCs in the tumor immune microenvironment and induce the inflammatory tumor microenvironment to exert a strong anti-cancer effect. It can also enable immune cells to produce proinflammatory and regulatory cytokines to produce anti-tumor effects, especially in combination with dendritic cell-based vaccines (Mitchell et al., 2021). R848 has been shown to mediate promising immunostimulatory activity in several preclinical models. Nanomedicine containing R848 for photothermal therapy was able to eradicate mouse 4T1 mammary tumors and prevent distant organ metastasis, suggesting a potential for combination with ICBs (Kim et al., 2016). Mouse pancreatic tumor responded to R848 with CD8^+^ T cells increasing and CD4^+^CD25+FOXP3+ regulatory T cells decreasing (Salmaso and Caliceti, 2013). Other TLR7 agonists such as MEDI9197 and DSP-0509 have been revealed to activate NK cells and inhibit polymorphonuclear MDSCs (Chen et al., 2020). Moreover, an ongoing clinical trial aiming at evaluating the HER2-targeted TLR7/TLR8 mixed agonist ISAC BDC-1001 is enrolling individuals with HER2^+^ breast cancer (NCT04278144). However, recent disappointing clinical test results reflect that the development of R848 as immunostimulatory agents used for cancer patients seems to stand at an impasse. Its hydrophobicity hinders its application *in vivo*. Efforts must be devoted to developing a delivery platform for R848 that can improve treatment efficiency through realizing stronger local activity without systemic exposure. As far as we are concerned, our study reports, for the first time, the combination of siCD47 with R848 for TNBC treatment. These results provide direct evidence for the feasibility of immunotherapy in combination with immune adjuvant to inhibit the growth of tumor while regulating the tumor invasion microenvironment.

Nanoparticles (NPs) have been identified as ideal carriers for diverse types of drugs (Sanchez-Moreno et al., 2018). Nanoparticles based on polylactic acid–glycolic acid (PLGA) approved by the FDA have wide application prospect due to their biocompatibility, biodegradability, storage stability, minimal systemic toxicity, and high bioavailability. However, application of PLGA NPs was limited because intravenous administration is rapidly cleared by the reticuloendothelial system. Thus, polyethylene glycol (PEG) has been conjugated with PLGA for the purpose of improving drug encapsulation efficiency and achieving long-term therapeutic effects. In addition, PEG-PLGA NPs improved surface hydrophilicity and enhanced the tumor-targeted accumulation by the EPR effect and improved its safety. Cancer gene therapy using nucleic acids cannot be extensively used in clinical practice as a result of their fast degradation and easy elimination in the blood (39). The utilization of R848 is also low due to its lipophilicity. Our NPs selected in this study consist of a PEG shell, and a PLGA core can effectively encapsulate hydrophilic CD47siRNA and hydrophobic R848. It provides a feasible strategy for delivering two kinds of drug molecules with different properties to tumor tissue *in vivo*. Although PLGA and PEG have been approved by the FDA as safe and biodegradable, few studies have explored the safety of PEG-PLGA NPs *in vivo* for further clinical application. Our study demonstrated that these NPs had few toxic and side effects in mice. We used the hydrophobic fluorescent dye DiD to verify that nanoparticles can effectively target cells, tissues, and organs and the duration in tumor site is longer even more than 24 h. Just as Salmaso (40) previously described, using the PEG-PLGA polymer could improve the stealth characteristics of drug nanocarriers and prolong the timeliness, which lays a foundation for the subsequent research of nanodrugs to play a role in inhibiting tumor growth. Using the NP/R848/siCD47 nanomedicine can safely and effectively inhibit tumor growth, which further confirmed the feasibility of Chen (41), Paola S á nchez Moreno (42), and other nanodrug carrying systems to carry drugs for treating malignant tumors. Therefore, the combination of CD47siRNA and R848 in the tumor can inhibit tumor proliferation, reflecting the synergistic effect of siCD47 blocking tumor immune escape and the R848-regulating immune infiltration microenvironment.

In summary, PEG-PLGA nanomaterials carrying CD47siRNA and R848 were prepared by using the double emulsification method. The caudal vein injection can effectively accumulate rapidly in the tumor and has good timeliness. At the same time, it can safely and effectively inhibit the growth of the tumor *in situ* by enhancing the autoimmune system. It provides a safe and effective new idea for the immunotherapy of triple-negative breast cancer and provides an effective research platform for the *in vivo* therapeutic effect study and mechanism exploration of nanodrugs for tumor immunotherapy by regulating the microenvironment of TNBC.

## Data Availability

The original contributions presented in the study are included in the article/Supplementary Material; further inquiries can be directed to the corresponding author.
