# Supplementary material for: Cationic lipid-assisted nanoparticles for simultaneous delivery of CD47 siRNA and R848 to promote antitumor immune responses
Source: Front Pharmacol. 2023 Mar 31;14:1142374. doi: 10.3389/fphar.2023.1142374 (PMC10102467; doi:10.3389/fphar.2023.1142374)
Supplement: Supplementary file 1 [file DataSheet1.docx]

**Supplementary Materials**

**
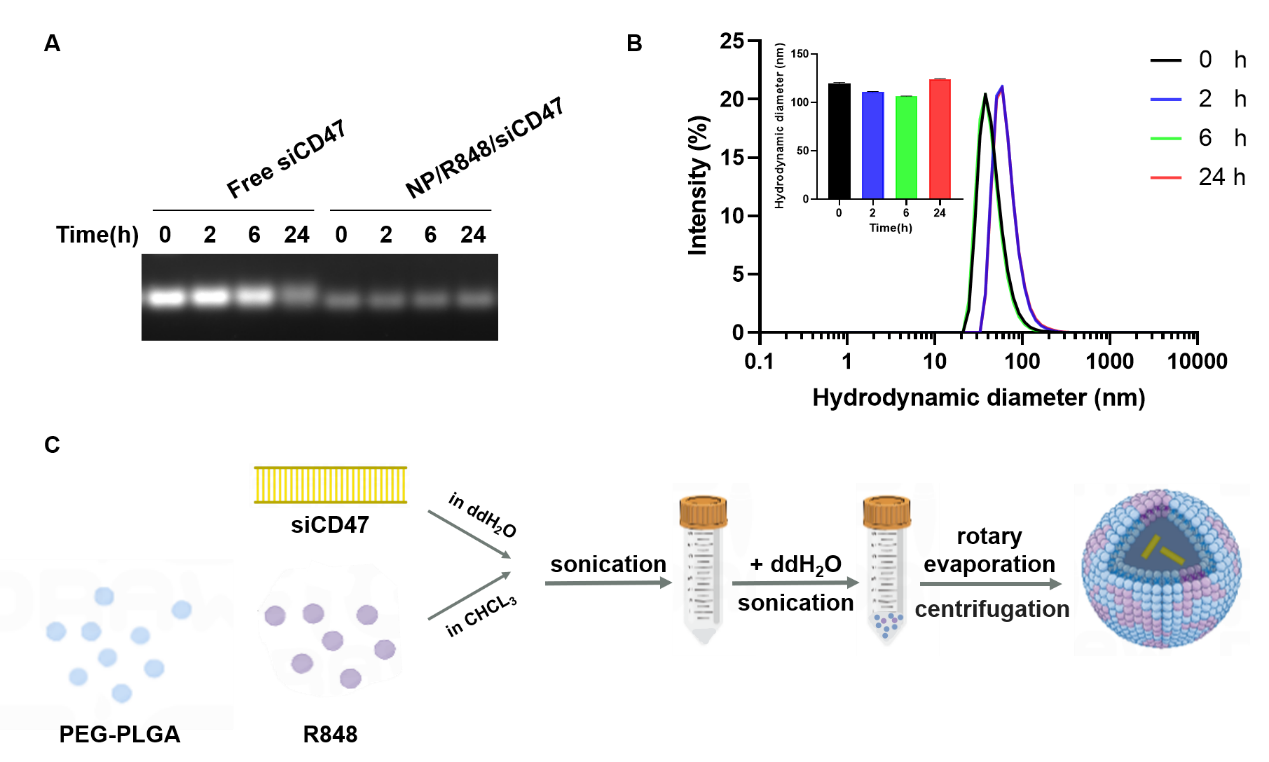
**

**Figure S1** (A) Susceptibility to serum-degradation of free or complexed siRNA within NP/R848/siCD47. The integrity of siRNA cargo was evaluated with an agarose gel electrophoresis assay. B) Intensity distribution of particle size of NP/R848/siCD47 after incubated in PBS containing 25% FBS at 37 ^o^C for 0, 2, 6, and 24 h. The diameter was measured by DLS (*n* = 3). (C) Preparation process of nanoparticles.

**
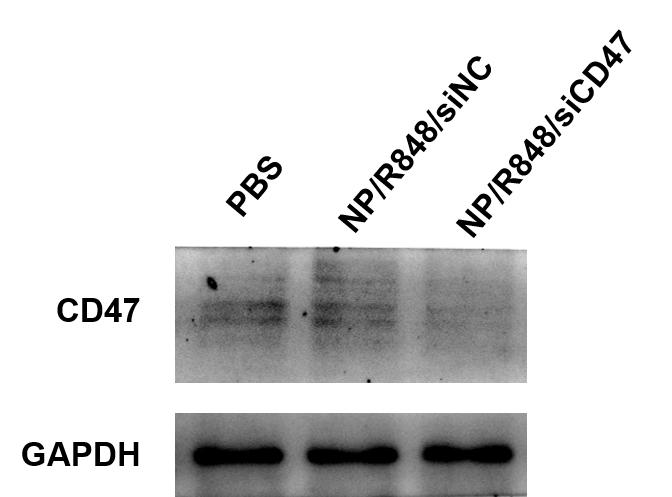
**

**Figure S2** Expression of CD47 proteins in 4T1 cells separately treated with PBS, NP/R848/siNC and NP/R848/siCD47 by Western blotting analysis.
